# Accuracies of genomic predictions for disease resistance of striped catfish to *Edwardsiella ictaluri* using artificial intelligence algorithms

**DOI:** 10.1093/g3journal/jkab361

**Published:** 2021-10-22

**Authors:** Nguyen Thanh Vu, Tran Huu Phuc, Kim Thi Phuong Oanh, Nguyen Van Sang, Trinh Thi Trang, Nguyen Hong Nguyen

**Affiliations:** 1 School of Science, Technology and Engineering, University of the Sunshine Coast, Sippy Downs, QLD, Australia; 2 Genecology Research Center, University of the Sunshine Coast, Sippy Downs, QLD, Australia; 3 Research Institute for Aquaculture No.2, Ho Chi Minh 710000, Vietnam; 4 Institute of Genome Research, Vietnam Academy of Science and Technology, Hanoi, Vietnam; 5 Vietnam National University of Agriculture, Gia Lam 131000, Vietnam

**Keywords:** striped catfish, *Pangasianodon hypophthalmus*, *Edwardsiella ictaluri*, BNP disease, genomic prediction, BayesR, machine learning and deep learning

## Abstract

Assessments of genomic prediction accuracies using artificial intelligent (AI) algorithms (*i.e.*, machine and deep learning methods) are currently not available or very limited in aquaculture species. The principal aim of this study was to examine the predictive performance of these new methods for disease resistance to *Edwardsiella ictaluri* in a population of striped catfish *Pangasianodon hypophthalmus* and to make comparisons with four common methods, *i.e.*, pedigree-based best linear unbiased prediction (PBLUP), genomic-based best linear unbiased prediction (GBLUP), single-step GBLUP (ssGBLUP) and a nonlinear Bayesian approach (notably BayesR). Our analyses using machine learning (*i.e.*, ML-KAML) and deep learning (*i.e.*, DL-MLP and DL-CNN) together with the four common methods (PBLUP, GBLUP, ssGBLUP, and BayesR) were conducted for two main disease resistance traits (*i.e.*, survival status coded as 0 and 1 and survival time, *i.e.*, days that the animals were still alive after the challenge test) in a pedigree consisting of 560 individual animals (490 offspring and 70 parents) genotyped for 14,154 single nucleotide polymorphism (SNPs). The results using 6,470 SNPs after quality control showed that machine learning methods outperformed PBLUP, GBLUP, and ssGBLUP, with the increases in the prediction accuracies for both traits by 9.1–15.4%. However, the prediction accuracies obtained from machine learning methods were comparable to those estimated using BayesR. Imputation of missing genotypes using AlphaFamImpute increased the prediction accuracies by 5.3–19.2% in all the methods and data used. On the other hand, there were insignificant decreases (0.3–5.6%) in the prediction accuracies for both survival status and survival time when multivariate models were used in comparison to univariate analyses. Interestingly, the genomic prediction accuracies based on only highly significant SNPs (*P* < 0.00001, 318–400 SNPs for survival status and 1,362–1,589 SNPs for survival time) were somewhat lower (0.3–15.6%) than those obtained from the whole set of 6,470 SNPs. In most of our analyses, the accuracies of genomic prediction were somewhat higher for survival time than survival status (0/1 data). It is concluded that although there are prospects for the application of genomic selection to increase disease resistance to *E. ictaluri* in striped catfish breeding programs, further evaluation of these methods should be made in independent families/populations when more data are accumulated in future generations to avoid possible biases in the genetic parameters estimates and prediction accuracies for the disease-resistant traits studied in this population of striped catfish *P. hypophthalmus*.

## Introduction 

Genomic selection has been increasingly practiced in genetic improvement programs for farmed animals and plants, using a range of different statistical methods from genomic-based best linear unbiased prediction (GBLUP) and its extension known as single-step GBLUP (ssGBLUP) to nonlinear Bayesian approaches (BayesA, BayesB, BayesC, BayesC-π, and notably BayesR) (VanRaden 2008; Daetwyler *et al.* 2013; Moser *et al.* 2015; Lourenco *et al.* 2020). Recently, there has been a growing interest in using artificial intelligent (AI) algorithms (*i.e.*, machine learning or deep learning) to choose optimal genome-wide models without prior consumptions to determine genomic prediction accuracies for quantitative complex traits, especially for disease resistance (tolerance or resilience). Examples using simulated and real animal and plant data showed that the prediction accuracies (*r*) using machine learning were 10% greater than linear (*i.e.*, GBLUP) and 1.3% greater than nonlinear methods (*i.e.*, BayesR), for instance, *r *=* *0.732–0.758 for binary and continuous traits using GBLUP *vs* 0.801–0.832 (Yin *et al.* 2020). Likewise, machine learning (*i.e.*, linear bagging) increased 20–70% the prediction accuracy for disease resistance to photobacterium in gilthead sea bream (Bargelloni *et al.* 2021). To date, however, no (or very limited) studies have used both machine and deep learning methods to estimate genomic breeding values for aquaculture species.

Almost all studies in aquatic animal species have employed GBLUP, ssGBLUP, or Bayesian methods to examine genomic prediction accuracies for two main groups of traits, *i.e.*, body weight and disease resistance. A synthesis of the published information shows that the genomic prediction accuracy ranged from 0.38 to 0.89 for growth-related traits (Houston *et al.* 2020). On the other hand, the predictions of genomic breeding values for disease resistance varied with studies, pathogens, populations, and methods used, with the estimates ranging from 0.2 (Silva *et al.* 2019) to 0.8 or greater (Barría *et al.* 2018). For both growth and disease traits, the majority of the studies demonstrated that either linear (GBLUP, ssGBLUP) or nonlinear Bayesian methods increased the prediction accuracies of animal breeding values relative to Pedigreed-based BLUP (PBLUP) by 22–24% (Houston *et al.* 2020). In addition to growth and disease traits, three recent studies performed genomic predictions for meat quality in banana shrimp (Nguyen *et al.* 2020) or Portuguese Oyster (*Crassostrea angulata*) (Vu *et al.* 2021) as well as behavior traits (*i.e.*, cannibalism) in Asian seabass (Nguyen and Khang 2021).

Despite the economic importance of the striped catfish in aquaculture, *e.g.*, valuing 2.36 billion USD that accounts for 1% GDP of Vietnam, there has been a paucity of knowledge in using genomic information in breeding programs for high growth (Vu *et al.* 2019b) or increased disease resistance, especially Bacillary Necrosis of Pangasius (Vu *et al.* 2019a). The Bacillary Necrosis of Pangasius (BNP) due to *Edwardsiella ictaluri* has been the main contributor to 56–92% mortality during larval and fingerling rearing in this species (Vũ *et al.* 2017). Our earlier studies showed that there is a heritable genetic component for *E. ictaluri* resistance, but the heritability for this trait was low, around 0.10 across statistical methods used (Vu *et al.* 2019a; Dinh Pham *et al.* 2021; Pham *et al.* 2021a, 2021b). While these results suggest there are prospects for genetic improvement of resistance to *E. ictaluri* using conventional selective breeding, the pathogen test has posed substantial challenges in terms of biosecurity issues, times, costs, and environmental impacts (Nguyen 2014; Dinh Pham *et al*. 2021). Due to these limitations, genomic selection has emerged as an alternative option to increase the resistance of striped catfish to *E. ictaluri*, one of the most severe diseases that has caused significant economic loss for the sector world-wide.

Therefore, the principal aim of this study was to assess genomic prediction accuracies of machine and deep learning methods for two main disease resistance traits (*i.e.*, survival status and survival time) and to make comparisons with four common methods (PBLUP, GBLUP, ssGBLUP, and BayesR). In addition, we explored if imputation of missing genotypes, multiple traits analyses and significant genome-wide markers could improve the prediction accuracies for the disease-resistant traits. Our results open new opportunities for genome-based selection to increase the animal resistance to *E. ictaluri* in striped catfish as well as other aquaculture species infected by this highly infectious pathogen.

## Materials and methods

### Ethical statement

All the methods and experimental protocols of this study were performed in accordance with guidelines and regulations approved by the animal ethics committee of the University of the Sunshine Coast, Australia (approval number ANE1826).

### Fish and challenge test

The animal samples used in this study originated from a selective breeding program for improved disease resistance of striped catfish to *E. ictaluri* (Vu *et al.* 2019a). In 2020, the first generation was produced based on a nested mating design with a ratio of one male to two females. A total of 166 families (32 full- and 134 half-sib families) were successfully produced following the breeding protocol as detailed in Van Sang *et al.* (2012) and Vu *et al.* (2019b). Fry of each family was kept in separate fiber glass tanks up to 3 weeks before they were transferred to stock in net hapa installed in earthen pond. When the fingerlings reached an average body weight of 15–20 g, a random sample of 100 fish per family was individually identified using passive integrated transponder (PIT) tag. After tagging, a half of each family was sent to ponds for performance testing and another half was used in pathogen challenge tests for *E. ictaluri* resistance.

The challenge test involved a total of 5328 individuals from 166 families (averaging 32 individuals per family). The experimental fish were initially acclimatized in cement tanks for about 2 weeks. Then the same number of fish from each family was randomly allocated to six cement tanks (10 m^3^) for the challenge test using cohabitant method (Vu *et al.* 2019a). The cohabitant fish (16.7 ± 6.1 g) were firstly inoculated with the bacteria *E. ictaluri* pathogen (10^6^ CFU/0.2 ml per fish). Two days after the injection, they were released into the cement tanks to rear with the experimental fish with a ratio of 1–3 (or roughly 30% cohabitant fish in each tank). The bacteria were added to the experimental tanks at day 4 at a dose of 10^5^ CFU/ml to retain the bacterial density for disease infection. The experiment was conducted over a period of 23 days when no death fish was recorded. During this period, the feeding rate was reduced to 1.5% of the total biomass in tank. Mortality was highest in day 5 and dead fish were sampled for laboratory PCR test to verify that their death symptoms (white spots in spleen, liver, and kidney) were due to *E. ictaluri* pathogen. At the conclusion of the experiment, all alive fish were biosecure-buried, following the regulations of the national veterinary authority (Department of Animal Health, Vietnam).

### Phenotype data

During the challenge test, death fish were collected every 3 h and their clinical symptoms were also recorded. The data were used to calculate two measures of *E. ictaluri* resistance, *i.e.*, survival status and survival time. Survival status was expressed as a binary trait in which dead fish were designated as zero (0) and alive animals at the end of the challenge test were assigned a number 1. Furthermore, survival time was defined as the continuous trait from the start of test until the animal death in day. Both survival status and survival time were analyzed using linear mixed model by ASReml 4.1 (Gilmour *et al.* 2014) to estimate breeding values (EBVs) for all individuals (5328) and families (166) in the pedigree, with common environmental effect—*c*^2^ (*i.e.*, accounting for differences due to separate rearing of families until tagging) fitted in the model. The linear mixed model comprised the fixed effects of spawning batch (7 levels) and challenge tanks (2 levels) and a covariate of age from birth to tagging. Estimated heritability and common full-sib effect obtained from this dataset using the above linear mixed models were 0.09 ± 0.06 and 0.06 ± 0.03 for survival status and 0.11 ± 0.08 and 0.13 ± 0.04 for survival time. Based on the EBVs ranking, 20 highest resistance families and 20 lowest resistance families were chosen from 166 families. Only families with the number of offspring greater than 9 were selected to ensure that the EBVs were estimated with a high level of reliability. Next, we randomly collected fin tissue samples of 12–15 fish per family per disease resistance group for genome sequencing using Diversity Arrays Technology (DArTseq^TM^).

### Genotyping and quality control

A total of 564 DNA samples (from 564 individual fish) were sent to a commercial service provider in Canberra, Australia for genotyping by sequencing using DArTseq^TM^ technology. The DArTseq^TM^ was based on the genome complexity reduction method in combination with high throughput next-generation sequencing using Illumina platform. The sequencing protocol including choice of restricted enzymes and library preparation was optimized for striped catfish (Pangasianodon DarTseq 1.0) using 96 independent DNA samples of striped catfish in our earlier study (Vu *et al.* 2020). Briefly, the PstI-SphI method (Kilian *et al.* 2012) was used and the compatible adaptors were designed to include Illumina flow-cell attachment sequence, sequencing primer sequence, and capturing variant length of barcode regions (Elshire *et al.* 2011). Then, only mixed fragments (PstI-SphI) were amplified in 30 rounds of PCR, followed by sequencing on Illumina Hiseq2500 (77 cycles per single read). Next, sequences generated in each plate with 96-well microliter were processed by proprietary DArT analysis protocol. The genotype callings were partially described in our previous studies (Nguyen *et al.* 2018, 2020). The average variant call-rate was 99% and the sample calling rate was 92%. With this quality control, four out of 564 samples were discarded, and 560 samples (490 offspring and 70 parents) and 14,154 single nucleotide polymorphisms (SNPs) remained for further quality control by dartR package (Gruber *et al.* 2018). In this data, average read depth in each locus ranged 2800–122,106 sequences, reads count from 1410 to 81,949 sequences. The quality control of the genotype data in the dartR package (Gruber *et al.* 2018) was filtered for loci call-rate (<0.05, 4665 SNPs removed) and individual call-rate (<0.9, 2 individuals removed), monomorphic SNPs (0 SNP removed), minor allele frequency (<0.05, 2830 SNPs removed) and significant SNPs departure from Hardy-Weinberg Equilibrium (<0.05, 0 SNP removed). The retaining 6,659 SNPs were blasted to the nonredundant nucleotide striped catfish genome database GENO_Phyp_1.0, https://www.ncbi.nlm.nih.gov/assembly/GCF_009078355.1 (Kim *et al.* 2018) which matched 6470 SNPs to the genome chromosome information using Blast2GO (Conesa *et al.*, 2005). The genotype data of 6470 SNPs and the phenotypes of 488 individuals were used for subsequent analyses.

### Genotype imputation

The missing genotypes were about 10.0% in this study. They were imputed using AlphaFamImpute (Whalen *et al.* 2020). The imputation was based on offspring-parent (*i.e.*, pedigree) information. Specifically, this method used parental genotypes to impute the missing values of offspring genotypes and then, the offspring genotypes were used to fulfill the missing values of their parents (Whalen *et al.* 2020). The pedigree used in our analysis was traced back to the base population, including four generations.

### Estimation of heritability using genotype data

Variances components of the two studied traits (survival status and survival time) were estimated using linear mixed model under the best linear unbiased prediction (BLUP) framework in AIREMLf90 sub-program of the BLUPF90 family package (Misztal *et al.* 2002). AIREMLf90 uses Average Information-Restricted Maximum Likelihood method that requires less computational resources and higher accuracy of variance estimates (Masuda *et al.* 2014). The models included the fixed effects of spawning batch (four levels, 1–2 weeks interval between successive spawning batches) and pathogen challenge test tanks (two levels) and the random effect of additive genetics of the individual fish in the pedigree. Age from birth to tagging (124–167 days) was also fitted as a linear covariate. The SNP heritability (*h*^2^) was calculated as a ratio of the additive genetic variance ($\sigma_{a}^{2}$) to total phenotypic variance ${(\sigma }_{p}^{2}$), where $\sigma_{p}^{2}$ = $\sigma_{a}^{2}$ + $\sigma_{e}^{2}$ ($\sigma_{e}^{2}$ = environmental variance). Based on the logarithmic likelihood ratio test (LRT), the common environmental effect (*c*^2^) was not significant (*P* > 0.05) and hence, this effect was omitted from the statistical models used to estimate genetic variances and prediction accuracy.

### Statistical methods

Accuracies of genomic prediction for the disease resistance traits (survival status and survival time) were estimated using BLUP-family methods (PBLUP, GBLUP, and ssBLUP), BayesR, machine learning (*i.e.*, ML-KAML), and deep learning (*i.e.*, DL-MLP and DL-CNN).

#### PBLUP, GBLUP, and ssGBLUP (using BLUPF90 program):

The mixed model used in PBLUP, GBLUP, and ssGBLUP is written in a matrix notation as follows:

(1) y=Xb+Za+e

where ***y*** is the vector of phenotypic values (survival status or survival time), ***b*** is the vector of fixed effects (*i.e.*, spawning batches and experimental tanks and a linear covariate of age from birth to tagging), ***a*** is the vector of the random term (*i.e.*, the additive genetic effect of individual fish in the pedigree). ***X*** and ***Z*** are the incidence matrices related to the fixed and random effects. The letter ***e*** refers to residual variance or error of the estimates corresponded to each phenotypic value.

The main difference among BLUP-family methods (PBLUP, GBLUP, and ssGBLUP) relates to relationship matrices (**A**, **G,** or **H**) used to solve the mixed model Equation (1) above.

**PBLUP:** In PBLUP (Henderson 1985), ***a*** is the additive genetic effect of each individual fish with its corresponding matrix **Z** following a normal distribution ∼N(0, **A**$\sigma_{a}^{2}$), with **A** is the numerator relationship matrix calculated from the pedigree records and $\sigma_{a}^{2}$ is the additive genetic variance.

**GBLUP**: In GBLUP (Meuwissen *et al.* 2001), ***a*** is the random additive genetic effect underlying polygenic effects of SNPs with a normal distribution ∼ N(0, **G**$\sigma_{g}^{2}$) where genomic relationship matrix **G** was generated by VanRaden method (VanRaden 2008) from 6470 SNPs and genomic variance $\sigma_{g}^{2}$. The GBLUP method assumes that each SNP has small and equal contribution to phenotypic variance of the traits studied.

**ssGBLUP**: In ssGBLUP (Misztal *et al.* 2009; Lourenco *et al.* 2020), **A** and **G** matrices are blended to produce realized matrix **H** (Aguilar *et al.* 2010). The inverse of H matrix is expressed as below:

(2) $$H^{-1}=A^{-1}+\left[\begin{array}{cc} 0 & 0\\ 0 & G^{-1}-A_{22}^{-1} \end{array}\right]$$

where ***A***^−1^ and ***G***^−1^ are the inverse of matrices ***A*** and ***G*** as described above. ***A***^−1^_22_ is the inverse of matrix of genotyped individuals only.

The three BLUP methods (PBLUP, GBLUP, and ssGBLUP) were implemented in BLUPF90 package (Misztal *et al.* 2002). For survival status, we used generalized (threshold) mixed model in THRGIBBF1f90 (Tsuruta and Misztal 2006) known as threshold Gibb Sampling method, whereas survival time was analyzed using linear mixed models in AIREMLf90 (Masuda *et al.* 2014). In the Gibbs sampling, we used 200,000 iterations with 20,000 iterations as burn-in for univariate analysis. The convergence of the parameters estimates was checked using POSTGIBBSf90 program.

#### Nonlinear Bayesian method (BayesR):

BayesR assumes that SNP effects *β_i_* follow a four-component normal mixture and the effect of SNP *i* is assumed to be distributed as:

(3) $$\beta_{i}\;∼\;\pi_{1}\left(\beta_{i}=0\right)+\pi_{2}N\left(0.0001\sigma_{g}^{2}\right)+\pi_{3}N\left(0,\;0.001\sigma_{g}^{2}\right)+\pi_{4}N\left(0,\;0.01\sigma_{g}^{2}\right)$$

where $\sigma_{g}^{2}$ as defined above, $\sigma_{g}^{2}$ represents the total additive genetic variance (*i.e.*, the cumulative variance of all SNP effects) and $\pi =(\pi_{1},\;\pi_{2},\;\pi_{3},\;\pi_{4})$ the mixing proportions such that $\sum_{i=1}^{4}\pi_{i}=1$. The mixing proportions π are assumed to follow a Dirichlet prior, π∼ Dirichlet (α+ β), with α representing a vector of pseudo counts and β the cardinality of each component. In this study, we used a flat Dirichlet distribution, with α=(1,1,1,1) for the prior. As suggested by Moser *et al.* (2015), $\sigma_{g}^{2}\;$is assumed to be a random variable following an Inverse—χ^2^ distribution.

The analysis of BayesR (https://github.com/syntheke/BayesR) was run for 50,000 steps with 20,000 steps discarded as burn-in. We used predicted phenotypes (computed in AIREMLf90 using Equation 1), as this package does not allow fitting covariates in the model. Also, genotypes executed in BayesR were converted using PLINK software (Purcell *et al.* 2007) together with mapping file in the previous step (see Section 2.3).

#### Machine learning and deep learning methods:

##### KAML (Kinship Adjusted Multiple Loci):

KAML is a machine learning-based method that incorporates cross-validation, multiple regression, grid search, and bisection algorithm to improve genomic prediction accuracy for complex quantitative traits (Yin *et al.* 2020). KAML extends linear mixed model (Equation 1) by incorporating quantitative trait nucleotides—QTNs (*e.g.*, traits controlled by genes with large and moderate effects) as covariates and a SNP-weighted trait-specific kinship matrix as the (co)-variance assumption corresponding to the random effect. The selections of QTNs and SNP weights are optimized by machine learning. In a matrix notation, Equation 1 becomes:

(4) $$Y=Xb+Qq+Za*+e\;\text{with}\;a*\;∼\;N(0,{\;K}_{w}\sigma^{2}{}_{g}),e∼\;N(0,\;I\sigma^{2}{}_{g})$$

where $\sigma_{g}^{2}\;$and $\sigma_{e}^{2}\;$are as described above; ***Q*** is covariates matrix of QTNs detected throughout multiple regression analyses; ***q*** is a vector of fixed effect of each QTN corresponding to ***Q*** and K_w_ is SNP-weighted Kinship that is optimized by grid search and bisection procedure. The implementation of KAML included two main steps, *i.e.*, define ***Q*** and K_w_ and then predicting genomic breeding values of the validation set. In the training step, KAML optimized parameters for determining QTNs and SNPs weights which used all testing subset data provided throughout cross-validation. Next, in the prediction step, the optimized parameters were used to predict genomic breeding values of each individual in validation set. A detailed description of the step-wise procedures and the underlying algorithms of KAML is given in Yin *et al.* (2020). In our analyses, we used PLINK (Purcell *et al.* 2007) to convert the genotype data into the right format to be analyzed in KAML (https://github.com/YinLiLin/KAML) in R environment (R Core Team 2015). KAML utilized adjusted phenotypes (*i.e.*, phenotypic y-hats) for testing sets and masked (*i.e.*, “NA” values) phenotypes of validation sets. To define the best parameters (*i.e.*, pseudo QTNs, the optimal SNPs weights, the optimal Log value and the optimized kinship matrix) for mixed models of each testing set, we used fivefold cross-validation with 100 replications (to obtain stable parameters over subsets, *i.e.*, QTNs and Log values).

##### Deep learning—Multilayer Perceptron (DL-MLP):

For deep learning analysis, we used DeepGP package (https://github.com/lauzingaretti/deepGP). A detailed description of the theoretical framework is given in Zingaretti *et al.* (2020) and Pérez-Enciso and Zingaretti (2019). In our analyses, we used two different algorithms of deep learning: multilayer perceptron and convolutional neural network.

Multilayer perceptron is fully connected networks consisting of an input layer, one or several hidden layers, and an output layer. In the context of genomic prediction, input layer receives SNPs data (*i.e.*, 6470 SNPs). Each of SNP information in the input layer is transferred its value to the first neuron of the first hidden layer by the Z function:

(5) $$Z^{\left(k\right)}=b_{k-1}+W^{\left(k-1\right)}f^{\left(k-1\right)}\left(x\right),$$

where *W* is weighted SNP information, *f(x)* is nonlinear function and *b* is bias (*i.e.*, constant). In the case of genomic prediction, the input layer is SNPs genotype (*i.e.*, input variables = number of SNPs) of individual fully connected to the first hidden layers. As a result, the value in the first neuron of the first hidden layer is the sum effect of Z function over 6470 SNPs ($\sum_{i=1}^{6470}Z$). Likewise, information in the first hidden layer is the input for transferring its value to the second hidden layers and finally to the output layers. The significance step in obtaining best DL-MLP model is to define MLP structure network (*i.e.*, number of hidden layer and neuron of each layer) and hyperparameters which related to Equation 5. These parameters and their values in our study include learning rate (–lr 0.025 0.01), type of drop out (–dr 0 0.01), type of regularization (–reg 0.0001), activation function (–act linear tanh), number of hidden layer (–h 1 5), optimizer function (–optimizer Adam) or number of epochs (–epochs 50). These scripts provided in DeepGP (https://github.com/lauzingaretti/DeepGP) were used to obtain hyperparameters in our DL-MLP analyses.

##### Deep learning—Convolutional Neural Network (DL-CNN):

Convolutional neural network comprises of 3 types of layers: (1) convolutional layers, (2) fully connected layers, and (3) input layers. In the context of genomic prediction, first convolutional layer receives data of 6470 SNPs and write it to the first layer using kernel (*i.e.*, a definite number of SNPs is grouped or combined). Then, the pooling step is responsible to reduce spatial size of SNPs data of the convolutional layer into the second convolutional layer. There are two types of pooling such as max pooling and average pooling corresponding to maximum values and mean values of SNPs portion covered by the kernel. The process was repeated to make update value of the last convolutional layer. At the last convolutional layer, data were flattened (*i.e.*, convert data of pooled feature of the last convolutional layer into single column feeding to fully connected layers). The fully connected layer works similarly the MLP. The hyperparameters used in our study are size of kernel (–ks 3), learning rate (–lr 0.025 0.01), dropout in the first layer (–dr1 0 0.01), dropout in the hidden layer (–dr2 0 0.1 0.2), number of pooling size (–ps 1), number of convolutional layers (–ncov 1 3), number of strides (–ns 1), number of convolutional operations (–nfilters 8). These hyperparameters are fine-tuned under DeepGP with grid search provided in DeepGP package (Zingaretti *et al.* 2020). As DeepGP cannot handle missing values, we used imputed genotypes for all analyses.

#### Analysis of highly significant SNPs and multi-trait mixed model:

##### ssGWAS to select significant SNPs for prediction:

Single step genome-wide association analysis (ssGWAS) was accomplished in BLUPF90 suite using each subset phenotype data (*i.e.*, see Section 2.7) for both disease resistance traits. Theoretically, there were seven steps in performing ssGWAS in BLUPF90 program (Lourenco *et al.* 2020). Practically, univariate model of BLUPF90 was used to estimate breeding values. The inverse of genomic relationship matrix generated with *xx_ija* file using “OPTION saveGInverse” and “OPTION snp_p_value” were read by postGSf90 to compute SNP effect and its *P*-values. SNPs panels were selected based on the *P*-value < 0.0001 (number of the selected SNPs sets were presented in Supplementary Table S2). These SNP panels were used to test the predictive performance of six genomic prediction methods used in our study.

##### Multi-trait mixed model:

Multi-trait analyses are only available in BLUPF90. In this study, two studied traits were jointly analyzed using PBLUP, GBLUP, and ssGBLUP models in THRGIBBS1F90 (Tsuruta and Misztal 2006). This involves using Gibbs Sampling with MCMC updates including 1,000,000 iterations with 100,000 burn-in steps.

### Fivefold cross validation

The predictive performances of all seven methods (PBLUP, GBLUP, ssGBLUP, BayesR, ML-KAML, DL-MLP, and DL-CNN) used in this study were evaluated using repeated fivefold cross validation. The procedure involved a random partitioning of the full data into 5 subsets (each subset has 97–98 individuals including representatives of all the families). The phenotypes of one subset were masked and GEBV/EBV (Genomic/Estimated Breeding Value) of this set were predicted using the information from the other four subsets. This means one phenotype was used once in the validation process of one repetition. Each analysis was repeated 5 times, giving 25 validation sets in total and the correlation coefficient was averaged over 25 values. To compute evaluation metric in each validation set, the GEBV/EBVs of the predicted phenotypes were correlated to the actual phenotype. We chose Pearson correlation coefficient (Benesty *et al.* 2009) as an evaluation metric to consistently compare the statistical models used. Moreover, we computed Mathew correlation coefficient, MCC (Matthews 1975) for binary trait (survival status) as MCC is giving a different look (indicate which predicted value is assigned to 0 or 1 case) for binary classification evaluation (Chicco and Jurman 2020). Specifically, we used *PresenceAbsence* R package (Freeman and Moisen 2008) to produce 4 × 4 confusion matrix which assigned GEBV/EBV values against the actual phenotypes for computing MCC (Supplementary Table S1 for additional information and reference). To obtain the consistency and comparable results among tested software/packages, we used the same subsets of testing/validation data (*i.e.*, 25 subsets) in BLUPF90 family program, BayesR, ML-KAML, DL-MLP, and DL-CNN.

The genomic prediction accuracy for survival status and survival time was calculated as $\mathrm{Accuracy}=\;\frac{r_{y,\hat{y}}}{\sqrt{h^{2}}};\;$where $r_{y,\hat{y}}$ is the correlation between the predicted breeding values $\left(\hat{y}\right)$ and actual phenotypes$\left(y\right)$. The heritability was estimated using pedigree-based analysis (PBLUP models).

To evaluate reliability (or potential biases) of the statistical methods, we computed root mean square error (RMSE) and *R*^2^ (coefficient of determination) based on $\hat{y}$ and y values. RMSE is the standard deviation of residuals, also known as prediction error, while coefficient of determination is an indicator of the goodness of fit of a statistical model. RMSE and R^2^ were calculated as below:

$$\mathrm{RMSE}=\sqrt{\frac{\sum_{1}^{n}{\left(\hat{y}-y\right)}^{2}}{n}}:\;R^{2}=1-\frac{\sum_{1}^{n}{\left(\hat{y}-y\right)}^{2}}{\sum_{1}^{n}{\left(y-\bar{y}\right)}^{2}}$$

where $\overset{-}{y}$ is the average value of *n* actual phenotypes in a testing set.

## Results

### Data, variance component, and heritability

**Figure 1:**
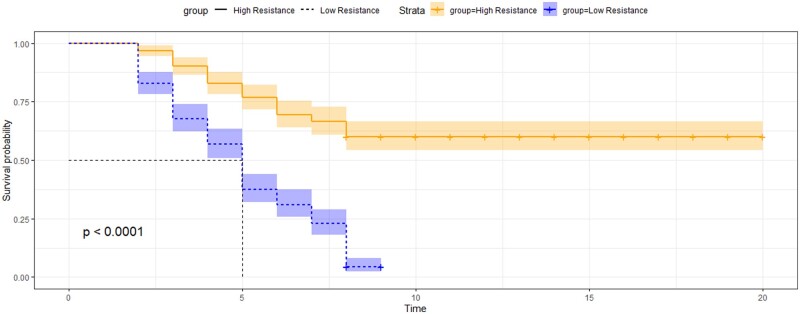
Survival trend in day post challenge of high and low resistance groups in genotyped individuals of striped catfish.

The average survival rate and survival time after the pathogen challenge test were 32.2% and 6.8 days, respectively (Table  1). When exposed to the *E. ictaluri* pathogen, mortality occurred after 1.4 days and the highest death rate was observed after 4.1 days (Figure  1). There were significant differences (*P* < 0.0001, Figure  1) in both survival rate (60.0% *vs* 4.5%) and survival time (5.0 *vs* 8.7 days) between 20 highest and 20 lowest resistant families (Table  1). The variations in the disease-resistant traits among families were also significant (Supplementary Figures S1 and S2). The results of our main challenge test experiment are provided in Supplementary Table S2.

**Table 1:** Number of animals (*n*) and phenotypes in the disease resistant and susceptible groups used for genotyping

Groups	20 lowest survival family		20 highest survival families		All 40 families	
Trait	Individuals	Mean ± SD	Ind/family	Mean ± SD	*n*	Mean ± SD
Survival status (%)	245	4.5 ± 20.8	245	60.0 ± 49.1	490	32.2 ± 46.8
Survival time (*d*)	245	5.0 ± 2.2	245	8.7 ± 4.3	490	6.8 ± 3.9

The heritabilities estimated for survival time and survival status using traditional PBLUP (0.65 ± 0.14 and 0.71 ± 0.14) were greater than those obtained from (ss)GBLUP (0.44 ± 0.09 and 0.46 ± 0.09) but they were lower than those estimated by BayesR (0.97 ± 0.02 and 0.96 ± 0.03) (Table  2). Across statistical models (methods) used, the estimates of heritability for survival status were slightly higher than those obtained for survival time (Table  2).

**Table 2:** Variance components and heritability for survival status and survival time using AIREMLf90 and THRGIBBS1f90 sub-program in BLUPF90 and BayesR

Trait	Models	Additive genetic variance	Phenotypic variance	Heritability
Survival status	PBLUP	0.147	0.207	0.71 ± 0.14
	GBLUP	0.086	0.188	0.46 ± 0.09
	ssGBLUP	0.088	0.190	0.46 ± 0.09
	BayesR	0.043	0.044	0.96 ± 0.03
Survival time	PBLUP	9.579	14.694	0.65 ± 0.14
	GBLUP	5.900	13.303	0.44 ± 0.09
	ssGBLUP	5.908	13.442	0.44 ± 0.09
	BayesR	2.747	2.818	0.97 ± 0.02

Note that estimate of genetic variance was not provided by ML-KAML, DL-MLP, and DL-CNN methods.

### Accuracy of genomic prediction using original genotype (un-imputed) data

**Figure 2:**
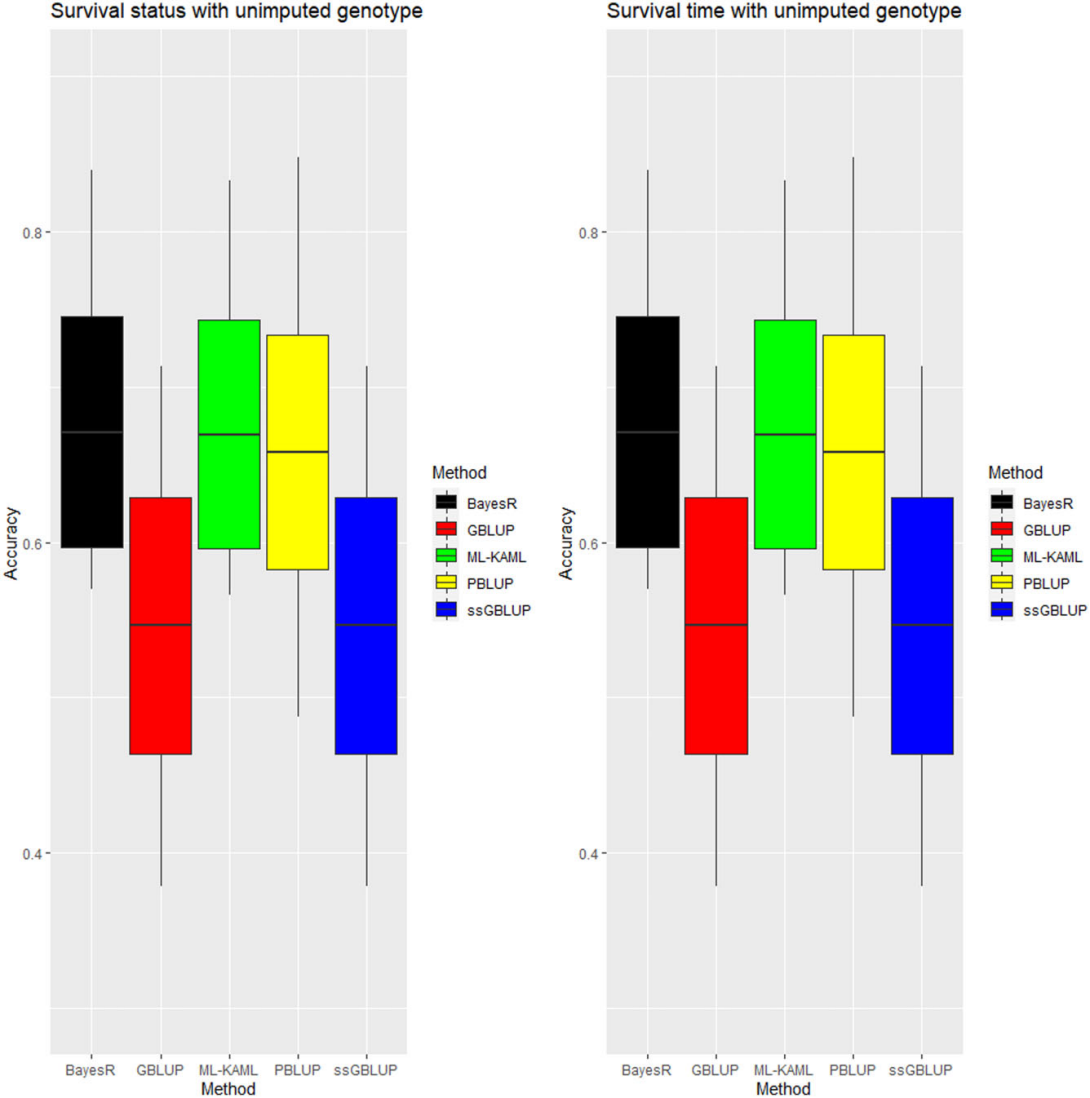
Accuracy of prediction for survival status and survival time traits using un-imputed 6,470 genotypes. Middle line of the box is mean accuracy; top and bottom lines of the box is accuracy ± one standard deviation. End points of vertical line represent min and max values. Note that PBLUP uses phenotype and pedigree information only.

Figure  2 (or Table  3) presents the accuracies of genomic breeding values for survival status and survival time using Machine learning (ML-KAML) together with four other methods (PBLUP, GBLUP, ssGBLUP, and BayesR). Note that DL-MLP and DL-CNN methods are not available for the analysis of missing genotypes. When the original genotype data (*i.e.*, un-imputed data) were used, the prediction accuracies from ML-KAML (0.67) were slightly higher than PBLUP (0.66) and they both were higher than those calculated from GBLUP (0.55) and ssGBLUP (0.55) for survival status. ML-KAML also outperformed BLUP methods for survival time (0.69 *vs* 0.59–0.65) (Table  3). In addition, the difference between ML-KAML and BayesR in the prediction accuracy was small (0.67 for survival status in both methods and 0.69 *vs* 0.70 for survival time). For both traits, ssGBLUP was slightly better GBLUP. Interestingly, both GBLUP and ssGBLUP did not improve the prediction accuracies for both survival status and survival time as compared with conventional PBLUP method.

**Table 3:** Accuracy between methods using nonimputed or imputed genotypes using 6470 SNPs

Trait	Method	Nonimputed		Imputed		Increasement (%)
		Accuracy ± sd (Pearson)	Accuracy ± sd (Mathew’s)	Accuracy ± sd (Pearson)	Accuracy ± sd (Mathew’s)	
Binary survival	PBLUP	0.66 ± 0.08	0.47 ± 0.11	—	—	—
	GBLUP	0.55 ± 0.08	0.39 ± 0.12	0.65 ± 0.07	0.51 ± 0.08	+19.0
	ssGBLUP	0.55 ± 0.08	0.39 ± 0.12	0.65 ± 0.07	0.51 ± 0.07	+19.2
	BayesR	0.67 ± 0.08	0.55 ± 0.09	0.76 ± 0.06	0.63 ± 0.09	+12.5
	ML-KAML	0.67 ± 0.07	0.57 ± 0.10	0.75 ± 0.06	0.63 ± 0.10	+12.0
	DL-MLP*	n.e.	n.e.	0.71 ± 0.11	0.65 ± 0.11	—
	DL-CNN	n.e.	n.e.	0.73 ± 0.08	0.63 ± 0.12	—
Survival time	PBLUP	0.65 ± 0.08	n.e.	—	—	—
	GBLUP	0.59 ± 0.09	n.e.	0.65 ± 0.09	n.e.	+10.0
	ssGBLUP	0.61 ± 0.07	n.e.	0.65 ± 0.09	n.e.	+7.1
	BayesR	0.70 ± 0.07	n.e.	0.75 ± 0.07	n.e.	+5.3
	ML-KAML	0.69 ± 0.07	n.e.	0.75 ± 0.07	n.e.	+8.1
	DL-MLP	n.e.	n.e.	0.67 ± 0.09	n.e.	—
	DL-CNN	n.e.	n.e.	0.70 ± 0.11	n.e.	—

n.e. not estimable due to DL-MLP and DL-CNN do not accept un-imputed missing genotype or not estimable for Mathew’s coefficient of continuous trait (i.e., survival time). Increasement (%) = (accuracy of imputed 6470 SNPs − accuracy of un-imputed 6470 SNPs)/accuracy of un-imputed 6470 SNPs

### Accuracy of genomic prediction using imputed genotype data

**Figure 3:**
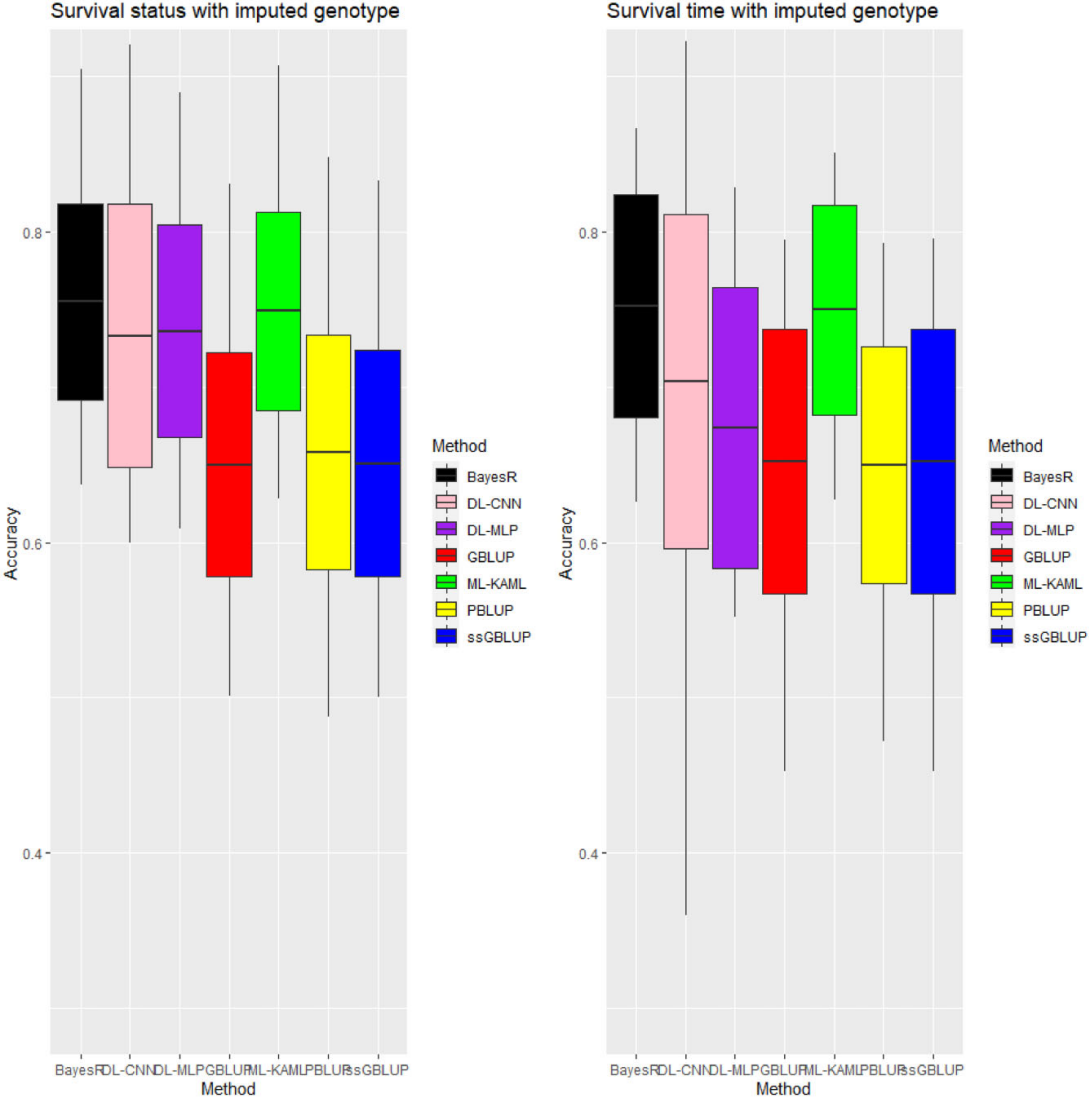
Accuracy of prediction for survival status and survival time traits using imputed 6,470 genotypes. Middle line of the box is mean accuracy; top and bottom lines of the box is accuracy ± one standard deviation. End points of vertical line represent min and max values. Note that PBLUP uses phenotype and pedigree information only.

Imputation of missing genotypes increased the accuracies of genomic prediction for both survival status and survival time across the four methods used, namely GBLUP, ssGBLUP, BayesR, and ML-KAML (Figure  3 or Table  3). The improvements for survival status were from 12.0% (ML-KAML) to 19.2% (GBLUP). For survival time, the increase in the prediction accuracy when using the imputed genotypes was largest for GBLUP (10.0%), followed by ML-KAML (8.1%), ssGBLUP (7.1%), and BayesR (5.3%). Deep learning (DL) using multilayer perceptron (DL-MLP) and convolutional neural network (DL-CNN) had lower prediction accuracies for both traits (0.67–0.73) than ML-KAML (0.75) or BayesR (0.75–0.76) methods but they (DL-MLP and DL-CNN) had higher prediction accuracies than BLUP methods (0.55–0.66) (Table  3). There were almost identical predictive performances between ML-KAML and BayesR methods for both traits regarding minimum, maximum, average, and standard deviation magnitudes of accuracy in all the 25 testing sets compared to other methods (Figure  3 or Table  3).

### Uni- *vs* multi-variate analysis

Our multivariate analyses used PBLUP, GBLUP, and ssGBLUP, and the prediction accuracies for both survival status and survival time are shown in Table  4. There were slight decreases observed in the prediction accuracy when the three BLUP methods were used to estimate genomic breeding values (GEBV) in multivariate analyses relative to univariate model. The reduction in the prediction accuracies was only 2.9–5.6% for survival time and 0.3–2.5% for survival status.

**Table 4:** Genomic prediction accuracy for survival status and survival time using univariate and bivariate models with un-imputed 6470 SNPs

Trait	Method	Univariate			Bivariate			Increasement (%)
		Accuracy ± sd	RMSE	*R* ^2^	Accuracy ± sd	RMSE	*R* ^2^	
Survival status	PBLUP	0.66 ± 0.08	0.39	0.30	0.66 ± 0.07	0.39	0.30	−0.3
	GBLUP	0.55 ± 0.08	0.41	0.21	0.53 ± 0.08	0.42	0.20	−2.5
	ssGBLUP	0.55 ± 0.08	0.41	0.21	0.54 ± 0.08	0.41	0.21	−0.7
Survival time	PBLUP	0.65 ± 0.08	3.30	0.26	0.63 ± 0.09	3.33	0.25	−2.9
	GBLUP	0.59 ± 0.09	3.41	0.22	0.57 ± 0.07	3.47	0.21	−3.5
	ssGBLUP	0.61 ± 0.07	3.38	0.23	0.58 ± 0.07	3.47	0.21	−5.6

Bivariate analysis is not available in BayesR, ML-KAML, Dl-MLP and DL-CNN. RMSE, root square mean error; *R*^2^, coefficient of determination; sd, standard deviation. Increasement (%) = (accuracy of bivariate—accuracy of univariate)/accuracy of univariate.

### Genomic prediction in combination with GWAS

The prediction accuracies for the disease resistance traits that incorporated only significant SNPs identified from genome-wide association analysis (GWAS) are given in Tables  5 and 6. When different genotype subsets were used (as tabulated in Supplementary Table S1), the prediction accuracies were generally smaller to those of the full set of 6470 SNPs. Using smaller SNP panels selected from ssGWAS with *P* < 0.00001 in this study scarified the prediction accuracies by 0.3–15.6%. For survival status, the prediction accuracy was reduced from 4.3% to 6.0% for ML-KAML and BLUP methods, but greater loss was observed for deep learning methods (11.2% for DL-CNN and 15.6% for DL-MLP). The same trend was observed for survival time but the reduction in the prediction accuracy was only 0.3–4.9%.

**Table 5:** Accuracy of genomic prediction using imputed genotypes of 6470 *vs* ssGWAS SNPs

Trait	Method	6,470 SNPs			ssGWAS SNPs			Increasment (%)
		**Accuracy** ± **sd**	RMSE	*R* ^2^	**Accuracy** ± **sd**	RMSE	*R* ^2^	
Survival status	GBLUP	0.65 ± 0.07	0.39	0.29	0.62 ± 0.07	0.40	0.26	−4.9
	ssGBLUP	0.65 ± 0.07	0.39	0.29	0.62 ± 0.07	0.40	0.27	−4.3
	BayesR	0.76 ± 0.06	0.36	0.39	0.71 ± 0.06	0.38	0.34	−6.0
	ML-KAML	0.75 ± 0.06	0.36	0.39	0.72 ± 0.06	0.37	0.36	−4.0
	DL-MLP	0.74 ± 0.08	0.37	0.38	0.62 ± 0.08	0.40	0.27	−15.6
	DL-CNN	0.73 ± 0.09	0.37	0.37	0.65 ± 0.07	0.46	0.35	−11.2
Survival time	GBLUP	0.65 ± 0.09	3.31	0.26	0.64 ± 12.5	3.31	0.25	−1.8
	ssGBLUP	0.65 ± 0.09	3.31	0.26	0.65 ± 11.4	3.30	0.26	−0.8
	BayesR	0.75 ± 0.07	3.11	0.35	0.75 ± 0.07	3.11	0.35	−0.3
	ML-KAML	0.75 ± 0.07	3.10	0.35	0.75 ± 0.07	3.10	0.35	−0.3
	DL-MLP	0.67 ± 0.09	3.25	0.29	0.64 ± 0.09	3.31	0.26	−4.9
	DL-CNN	0.70 ± 0.11	3.20	0.30	0.69 ± 0.07	3.25	0.30	−2.1

6470 SNPs were filtered with dartR packages. ssGWAS SPs were selected based on *P*-value of each SNPs effect (<0.00001) in each testing set. RMSE, root mean squared error and coefficient of determination; sd, standard deviation. Increasement (%) = (accuracy of ssGWAS SNPs − accuracy of 6470 SNPs)/accuracy of 6470 SNPs.

**Table 6:** Accuracy (±sd) of genomic prediction for survival status and survival using highly significant SNPs (*P* < 0.0001)

Trait	Method	Original (un-imputed) genotype			Imputed genotype		
		6470 SNPs	ssGWAS SNPs	Increase mnt (%)	6470 SNPs	ssGWAS SNPs	Increase mnt (%)
Survival status	PBLUP	0.66	—	—	—	—	—
	GBLUP	0.55	0.48 ± 0.09	−12.5	0.65	0.62 ± 0.07	−4.9
	ssGBLUP	0.5	0.49 ± 0.09	−9.9	0.65	0.62 ± 0.07	−4.3
	BayesR	0.67	0.62 ± 0.09	−8.2	0.76	0.71 ± 0.06	−6.0
	ML-KAML	0.67	0.61 ± 0.09	−8.5	0.75	0.72 ± 0.06	−4.3
	DL-MLP	—	—	—	0.74	0.62 ± 0.08	−15.6
	DL-CNN	—	—	—	0.73	0.65 ± 0.07	−11.2
Survival time	PBLUP	0.65	—	—	—	—	—
	GBLUP	0.59	0.59 ± 0.12	+0.2	0.65	0.64 ± 0.13	−1.8
	ssGBLUP	0.61	0.60 ± 0.12	−0.3	0.65	0.65 ± 0.11	−0.7
	BayesR	0.71	0.70 ± 0.07	−2.1	0.75	0.75 ± 0.07	−0.4
	ML-KAML	0.69	0.70 ± 0.06	+ 0.3	0.75	0.75 ± 0.07	−0.3
	DL-MLP	—	—	—	0.67	0.64 ± 0.09	−4.9
	DL-CNN	—	—	—	0.70	0.69 ± 0.07	−2.1

Sd, standard deviation; increasement (%) = (accuracy of ssGWAS SNPs − accuracy of 6470 SNPs)/accuracy of 6470 SNPs using unimputed or imputed genotypes.

### Reliability (or potential biases) of the statistical methods

The RMSE and coefficient of determination (*R*^2^) were computed for each method and they are shown in Tables  4 and 5. In both traits (survival status and survival time) and in all analyses (univariate and multi-trait or full genotype *vs* subsets), the machine learning (*i.e.*, ML-KAML) gave the least possible biases in the breeding values prediction, as evidenced by the low RMSE (0.36–0.37 for survival status and 3.10 for survival time) and high *R*^2^ (0.35–0.39). BayesR also displayed similar predictive reliability to ML-KAML (RMSE = 0.36–0.37 for survival status and 0.311 for survival time and *R*^2^ = 0.34–0.39). Among BLUP family methods, there were no large differences in the reliability of the genomic breeding value estimation between GBLUP and ssGBLUP (for instance with univariate model using un-imputed genotype, RMSE = 0.41 and *R*^2^ = 0.20–0.21). However, they were likely less reliable than PBLUP (RMSE = 0.39 and *R*^2^ = 0.30). The utilization of the imputed genotypes in GBLUP and ssGBLUP methods reduced the prediction errors for both survival status and survival time (RMSE = 0.39 and 3.31, and *R*^2^ = 0.29 and 0.26, respectively).

## Discussion

Development of disease resistance lines is essential for the aquaculture sector due to the combined effects of aquaculture intensifications and environmental changes. Our study demonstrated that genomic selection can be practiced improving resistance to *E.* *ictaluri* disease in striped catfish populations, using a range of different statistical methods especially machine and deep learning, and BayesR. However, further evaluation of these methods should be made in randomly selected samples or independent families/populations to avoid any possible biases in the genetic parameters estimates and prediction accuracies for the two traits studied. Salient findings from our study are discussed as follows.

First, the genomic prediction accuracies for the disease resistance traits (survival status and survival time) were substantially improved when machine (*i.e.*, ML-KAML) and deep learning (DL-MLP and DL-CNN) methods were used relative to BLUP-family methods (PBLUP, GBLUP, and ssGBLUP). Interestingly, PBLUP outperformed (ss)GBLUP methods in our study (Figure  2), which was rarely reported in the literature as reviewed by Houston *et al.* (2020). This implies that the SNP assumption of (ss)GBLUP does not reflect the true SNPs’ distribution as the AI and BayesR methods, at least in our data. In addition, our evaluation of the prediction biases or prediction errors based on RMSE and R^2^ statistics indicated that the AI methods may be subject to the least errors in the estimated breeding values than BLUP family methods. Hence, genomic selection using AI and BayesR methods to improve *E. ictaluri* disease is expected to achieve greater genetic gain than the conventional selective breeding approach using pedigree and phenotype information. The genome-based selection enables early recruitment of breeding candidates and hence, potentially reducing generation time in striped catfish as expected to reduce at least a half of generation interval in dairy cattle (Hayes *et al.* 2009). Our results are consistent with those reported in farmed animal (Baker *et al.* 2020), livestock (González-Recio and Forni 2011; Li *et al.* 2018), and plants (Montesinos-López *et al.* 2018, 2019; Zingaretti *et al.* 2020). Despite the superiority of the machine learning methods (*i.e.*, ML-KAML) to PBLUP, GBLUP and ssGBLUP, they had similar predictive power to BayesR for both traits in our study. Reports in farmed animals concluded that BayesR is the best amongst Bayesian and GBLUP methods (Erbe *et al.* 2012) but no published information is available in aquaculture species to compare with our study. Previous studies indicated other Bayesian methods (*i.e*., BayesB) outperformed PBLUP and (ss)GBLUP (Sukhavachana *et al.* 2020; Joshi *et al.* 2021) while GBLUP was similar to BayesA and BayesCπ for predicting nine traits of Portuguese oyster (Vu *et al.* 2021). To date, studies using machine and deep learning, one in shrimp (Palaiokostas 2021) that applied extreme gradient boost method (one of the machine learning approaches) and another in seabream that used support vector machines and linear bagging classification (Bargelloni *et al.* 2021) and reported that these methods improved the prediction accuracy for survival traits by 1–4% and 20–70% relative to GBLUP and Bayesian methods. Recently, the evaluation of deep learning model (*e.g.*, convolutional neural network) in Bay scallop indicated this method outperformed BayesB and random regression GBLUP methods (Zhu *et al.* 2021). Therefore, machine or deep learning should be used for genomic evaluation of disease resistance traits, at least in this population of striped catfish. The predictive powers of these methods using AI algorithms are expected to be greater (Montesinos-López *et al.* 2021) when big data (*e.g.*, hundreds of thousands or million animals sequenced) are analyzed for this population in the future.

Second, imputation of missing genotypes (using offspring and parent information available in the pedigree of striped catfish) increased the predictive ability of all the seven methods used, especially BayesR by 5.3%–approximately 19% (GBLUP and ssGBLUP) relative to when un-imputed data were used. Also note that the imputation of missing genotypes in our study differed from others that used software to compute missing genotypes without considering parental relationship or shared haplotype in full-sib families, *e.g.*, Beagle (Browning and Browning 2009) or AlphaPeel (Whalen *et al.* 2018). With the use of offspring-parent information such as AlphaFamImpute (Whalen *et al.* 2020) or FImpute (Sargolzaei *et al.* 2014), the imputation likely increased accuracy of G and H matrices used in GBLUP and single-step methods (*i.e.*, ssGBLUP) (Hickey *et al.* 2012). Published information across species also showed that imputation from medium to high-density SNP panels (25–78k SNPs) increased the genomic prediction accuracies for resistance to parasite (*i.e.*, sea lice count) and body traits in Atlantic salmon (Tsai *et al.* 2017), or from commercial SNP arrays to whole genome sequence improved the accuracies for agronomic traits in maize (Crossa *et al.* 2013). However, some other studies also showed that imputed genotype did not improve prediction accuracies in livestock animal breeding (Chen *et al.* 2014; Van Binsbergen *et al.* 2015; Heidaritabar *et al.* 2016). Imputation has several benefits, first it can be made after sequencing with no extra cost. Second, through imputation, we can increase the number of animals genotyped and the number of breeding candidates, which in turn increase selection intensity, and this can lead to increases in genetic progress made in selected populations (Gorjanc *et al.* 2017). Thus, imputation of low-density sequence can support our future incorporation of genomic selection for *E.* *ictaluri* disease in this striped catfish population.

Third, we found that multivariate analysis did not improve the predictive ability for the two studied traits irrespective of the statistical methods. These results are not expected, despite the high and positive genetic correlations (*r_g_*) between the two traits (*r_g_* = 0.81). A bivariate analysis of harvest body weight and fillet yield in Nile tilapia also showed no improvement in the prediction accuracies for these traits (Joshi *et al.* 2020). Multi-trait analyses generally improve accuracy of genetic parameter estimates and animal breeding values for traits whose genetic architectures are different (*e.g.*, low *vs* high heritable) or genetically antagonistic associated (*i.e.*, unfavorably correlated). Examples of multivariate genomic prediction models involving multi-trait in cassava and wheat plants gave better prediction accuracy over univariate models (Okeke *et al.* 2017; Sun *et al.* 2017). To utilize the benefits of multi-trait analysis, we continue collecting other traits of economic importance in striped catfish, namely disease tolerance, disease resilience as well as immunological parameters to increase overall resistant capacity of the animals in this catfish population.

Fourth, we suggest using the full set of SNPs to obtain a reasonable level of prediction accuracy for genomic selection to improve disease resistance traits in striped catfish. It has been well documented that the prediction accuracy increased with number of markers and sequencing depth or genome coverage (Pook *et al.* 2021). Due to characteristics of RAD-sequencing methods, there are still limited number of markers in our study as compared with those reported in farmed animals or plants (Davey *et al.* 2010; Robledo *et al.* 2018). It is necessary to examine prediction ability of whole-genome data when sequencing costs are reduced to an affordable level to sequence thousands (or hundreds of thousands) of animals in a near future. However, there are studies in farmed animals reporting that whole-genome sequence data improved the predictive powers for complex traits by only 0.4–5.0% (VanRaden *et al.* 2017; Al Kalaldeh *et al.* 2019).

Finally, the utilization of highly significant SNPs from ssGWAS can give comparative prediction accuracies for the two traits studied, for both un-imputed and imputed genotype data, as compared to the full set of 6470 SNPs in our present study. However, previous reports showed that using significant SNP panels selected from ssGWAS had higher accuracy in the estimated breeding values for disease resistance traits in *Litopeneaus vannamei* (Luo *et al.* 2021) or fish species (Jeong *et al.* 2020). On the one hand, these SNPs can be used to develop a small SNP panel to genotype a large number of animals in both the training and validation populations. On the other hand, the costs of genotyping animals for hundreds of SNPs may not be a lot cheaper than DArT sequencing as used in this study. The use of significant SNPs can be beneficial when their biological functions (pathways) are known and included in genomic prediction models. In species where reference genome or high-quality genome assembly is available, the incorporation of SNPs with biological functions (*i.e.*, multi-omics) improved the prediction accuracy, for instance, about 5–19% for fertility traits in dairy cattle (Abdollahi-Arpanahi *et al.* 2017; Nani *et al.* 2019), or 27.4–60.7% for traits in inbred lines of Drosophila (Ye *et al.* 2020). Unfortunately, a good genome assembly is currently not available for striped catfish (Kim *et al.* 2018), this area thus deserves future studies to enable the efficient utilization of genomic information in the genetic selection program for this economically important species in the aquaculture sector.

In summary, despite the high level of consistency in the prediction accuracies across the seven methods used, the estimates of heritability and prediction accuracies obtained for survival status and survival time may be potentially biased upward (*e.g.*, the high heritability from BayesR), likely due to the family structure of the current population that included selective genotypes (high and low resistant families). However, this problem may have been alleviated because both pedigree and genotype data were included in mixed models (*i.e.*, BLUP family and machine learning methods) to estimate genomic breeding values as described in Gowane *et al.* (2019). When more genotype and phenotype data and full pedigree records are collected, further analyses should be conducted to avoid possible biases in the prediction accuracies for the disease-resistant traits in this population of striped catfish *Pangasianodon* *hypophthalmus*.

## Concluding remarks

The genomic prediction accuracies for disease resistance traits of striped catfish were moderate to high, suggesting possibilities for the application of genomic selection to improve resilience to *E.* *ictaluri* in this population. Machine learning methods outperformed BLUP-family in almost all our analyses. However, the prediction accuracies using machine learning methods for both survival status and survival time were almost similar to those obtained from BayesR. Therefore, either machine learning, deep learning or BayesR could be used for genomic evaluation of the disease resistance traits in this striped catfish population. Furthermore, breeding to improve resistance to *E. ictaluri* can use survival status or survival time as alternative selection criterion as there was no significant difference in the prediction accuracies across the seven different methods used in our study. However, when more data are accumulated in future generations, it is necessary to re-evaluate the prediction accuracies and potential biases of these methods before any practical implementation of genomic selection can be made to improve the disease resistance of this striped catfish population.

## Data availability

All data are available via Figshare portal at https://doi.org/10.25387/g3.16713361. These comprised of full phenotypic, randomized subsets (*i.e.*, real and yhats), original dart-seq genotypes, and pedigree information.
